# Determining the Reliable Measurement Period for Preoperative Baseline Values With Telemonitoring Before Major Abdominal Surgery: Pilot Cohort Study

**DOI:** 10.2196/40815

**Published:** 2022-11-28

**Authors:** Marjolein E Haveman, Rianne van Melzen, Mostafa El Moumni, Richte C L Schuurmann, Hermie J Hermens, Monique Tabak, Jean-Paul P M de Vries

**Affiliations:** 1 Department of Surgery, Division of Vascular Surgery University Medical Center Groningen University of Groningen Groningen Netherlands; 2 Department of Anesthesiology University Medical Center Groningen University of Groningen Groningen Netherlands; 3 Department of Surgery, Division of Trauma Surgery University Medical Center Groningen University of Groningen Groningen Netherlands; 4 Department of Biomedical Signals and Systems University of Twente Enschede Netherlands; 5 eHealth group Roessingh Research and Development Enschede Netherlands

**Keywords:** telemonitoring, major abdominal surgery, preoperative, wearable sensor, vital signs, patient-reported outcome measure, PROM, surgery, major surgery, abdominal surgery, observational study, pain, anxiety, fatigue, nausea, heart rate, step count

## Abstract

**Background:**

Preoperative telemonitoring of vital signs, physical activity, and well-being might be able to optimize prehabilitation of the patient’s physical and mental condition prior to surgery, support setting alarms during in-hospital monitoring, and allow personalization of the postoperative recovery process.

**Objective:**

The primary aim of this study was to evaluate when and how long patients awaiting major abdominal surgery should be monitored to get reliable preoperative individual baseline values of heart rate (HR), daily step count, and patient-reported outcome measures (PROMs). The secondary aim was to describe the perioperative course of these measurements at home.

**Methods:**

In this observational single-center cohort study, patients used a wearable sensor during waking hours and reported PROMs (pain, anxiety, fatigue, nausea) on a tablet twice a day. Intraclass correlation coefficients (ICCs) were used to evaluate the reliability of mean values on 2 specific preoperative days (the first day of telemonitoring and the day before hospital admission) and randomly selected preoperative periods compared to individual reference values. Mean values of HR, step count, and PROMs per day were visualized in a boxplot from 14 days before hospital admission until 30 days after surgery.

**Results:**

A total of 16 patients were included in the data analyses. The ICCs of mean values on the first day of telemonitoring were 0.91 for HR, 0.71 for steps, and at least 0.86 for PROMs. The day before hospital admission showed reliability coefficients of 0.76 for HR, 0.71 for steps, and 0.92-0.99 for PROMs. ICC values of randomly selected measurement periods increased over the continuous period of time from 0.68 to 0.99 for HR and daily step counts. A lower bound of the 95% CI of at least 0.75 was determined after 3 days of measurements. The ICCs of randomly selected PROM measurements were 0.89-0.94. Visualization of mean values per day mainly showed variable preoperative daily step counts (median 2409, IQR 1735-4661 steps/day) and lower postoperative daily step counts (median 884, IQR 474-1605 steps/day). In addition, pain was visually reduced until 30 days after surgery at home.

**Conclusions:**

In this prospective pilot study, for patients awaiting major abdominal surgery, baseline values for HR and daily step count could be measured reliably by a wearable sensor worn for at least 3 consecutive days and PROMs during any preoperative day. No clear conclusions were drawn from the description of the perioperative course by showing mean values of HR, daily step count, and PROM values over time in the home situation.

## Introduction

The use of telemonitoring has been associated with improved clinical outcomes and cost-effectiveness of care in several fields of medicine [1,2]. Telemonitoring may be of great value in the preoperative phase, where telemonitoring at home may give a good indication of patients’ individual baseline values, such as vital signs, physical activity, and the level of experienced pain and anxiety [3,4]. This information is expected to assist in clinical decision-making (ie, by risk assessment), optimize prehabilitation of the patient’s physical and mental condition prior to surgery [3], support setting alarms during in-hospital monitoring, and allow personalization of the postoperative recovery process.

Despite these potential advantages, vital signs, physical activity, and patient-reported outcome measures (PROMs) of patients undergoing major abdominal surgery are not routinely monitored at home. Preoperative vital signs are often only measured in-hospital as part of the preoperative anesthetic screening and at hospital admission prior to surgery. Disadvantages of current practice are preoperative assessments being labor intensive and performed up to 12 weeks before surgery [5], and measurements during admission potentially being less representative because of increased psychological stress. Only a few studies describe baseline values before major abdominal surgery by telemonitoring at home. These studies mainly investigated the association between preoperative physical activity level and postoperative complications, readmissions, or functional recovery as a percentage of baseline values at 2 to 30 days before surgery [3,6-8]. It is currently unknown what period is sufficient to measure reliable baseline values for vital signs, steps, or PROMs in the time period that patients are on the waiting list for major abdominal surgery.

The primary aim of this study is to evaluate when and how long patients should be monitored at home to get reliable preoperative individual baseline values of heart rate (HR), step count, and PROMs (pain, anxiety, fatigue, nausea) before major open abdominal surgery. The secondary aim was to describe the course of HR, step count, and PROMs measured by telemonitoring at home before and after major abdominal surgery. This study was part of a prospective pilot study to evaluate the feasibility and patient experiences with perioperative telemonitoring (published separately [9]) to inform future study design.

## Methods

### Ethics Approval

The ethical committee of the University Medical Center Groningen approved the protocol (Telemonitoring in the Peri-operative Phase of Patients Undergoing Open Abdominal Surgery in a University Medical Center: A Pilot Study [PROMISE-study], research register number #201900432), and the study was conducted in accordance with the STROBE (Strengthening the Reporting of Observational Studies in Epidemiology) guidelines [10] and the Declaration of Helsinki.

### Study Design and Participants

Between January 2020 and January 2021, a prospective observational cohort pilot study was performed at the University Medical Center Groningen, a large tertiary referral hospital in The Netherlands.

Patients were recruited if they were planned for elective major open abdominal surgery (vascular, hepato-pancreato-biliary, or lower gastrointestinal) at the outpatient clinic based on procedure codes in the electronic health record during the aforementioned study period. Eligible patients were expected to be on the waiting list for at least 2 weeks and have access to Wi-Fi at home. Exclusion criteria were being mentally incapable of participation, not able to walk without an aid, or unable to wear a sensor on the upper arm. The sample size for this pilot study was set at 20 patients. Study participation of a patient was paused if surgery was significantly delayed or ended if surgery was cancelled, or if a patient had severe postoperative complications.

### Telemonitoring

After giving informed consent, patients received the telemonitoring devices and instructions at home from one of the executing researchers (MEH and RvM). The telemonitoring devices consisted of a wearable sensor (Everion, Biovotion AG, Zürich, Switzerland) and a tablet (Samsung Galaxy Tab A 10.1 2019). Patients were instructed to wear the sensor on the upper arm of their choice during waking hours and charge it during the night (sensor battery life was up to 40 hours, which required charging every 24 hours in practice). The Everion is a CE class IIa-certified wearable sensor that monitors vital signs based on photoplethysmography and physical activity (ie, step count) using an accelerometer with a sampling frequency of 1 Hz for vital signs and activity (raw data mode 51.2 Hz). The storage frequency for vital signs was once per minute and once per hour for step count. Data were transferred to the HealthyChronos application (HealthyChronos, Alphen aan den Rijn, Netherlands) on the tablet through Bluetooth and to the in-hospital database using Wi-Fi.

Based on previous validation studies with the Everion sensor [11,12], only HR was considered in this study. It has been shown that Everion underestimated HR by up to 5.3 beats per minute (bpm) and had a median absolute percentage error of 2.3% during daily activities compared to Holter measurements in volunteers [11]. Besides, HR had a moderate relationship (*r*=0.52) with nurse measurements in the surgical ward [12]. Respiration rate, blood oxygen saturation, and skin temperature measured by Everion had lower reliability and accuracy during daily activities in volunteers [11], and a poor relationship with nurse measurements in surgical patients [12]. To our best knowledge, the accuracy of Everion for daily step count is still unknown.

Patients received a notification to report PROMs twice a day: once in the morning (at random between 9 AM and 1 PM) and once at 8 PM in the mobile app on the tablet running on the Roessingh Research and Development eHealth platform (Activity Coach, Roessingh Research and Development, Enschede, The Netherlands [13]). PROMs included pain, anxiety, nausea, and fatigue on a visual analog scale (VAS) from 0 (no pain, anxiety, etc) to 10 (worst pain, anxiety, etc) imaginable.

Since this was an observational study without intervention, patients, and health care personnel were blinded to the telemonitoring data, and they did not receive feedback from the used technology.

### Data Selection

**Figure 1:**
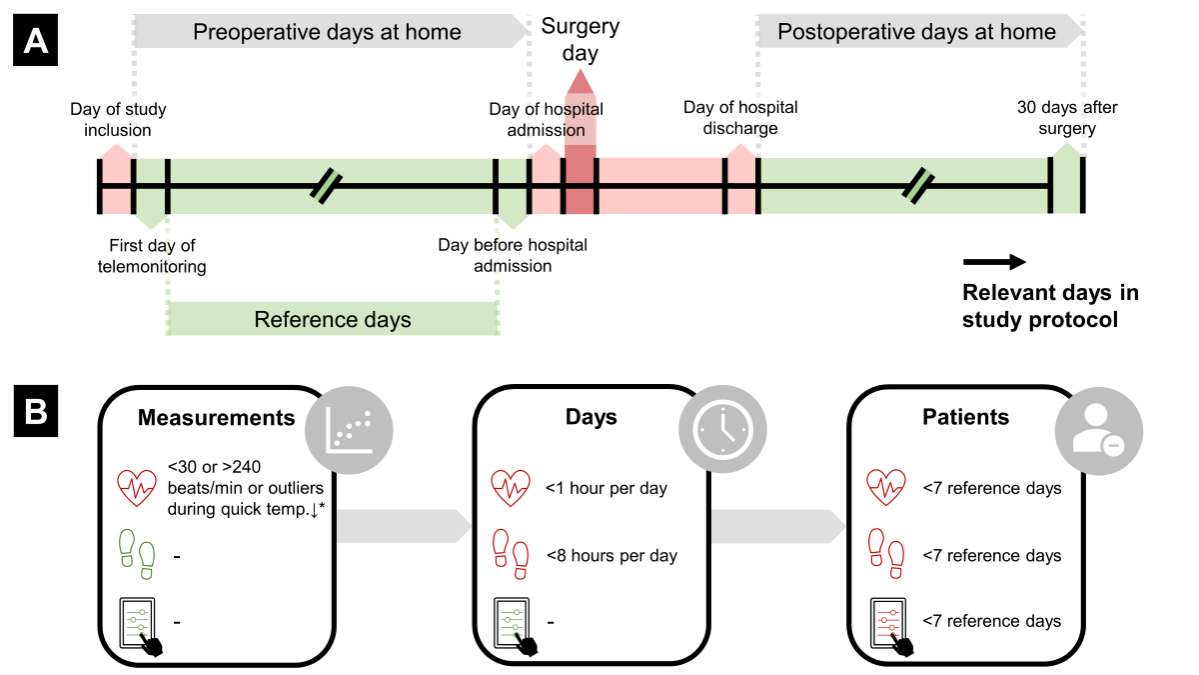
Schematic overview of (A) included (green) and excluded (red) preoperative and postoperative days at home, and (B) exclusion criteria at the level of measurements, days, and patients per parameter: heart rate, daily step count, and patient-reported outcome measures. See text for further explanation of measurement error removal.

Outcome measures were continuous data of HR and step count measured with the wearable sensor, and PROMs reported in the mobile app on the tablet, both preoperatively and postoperatively at home. Figure 1A shows a schematic overview of the preoperative and postoperative periods.

Data from telemonitoring were retrieved from the databases and processed and analyzed in Matlab R2021b (Mathworks, Inc). To minimize bias in statistical analyses, the research team defined exclusion criteria at the level of measurements, days, and patients per parameter, as shown in Figure 1B. First, measurements were excluded if they met one of the following criteria [12]: (1) if HR was measured outside the technical ranges as stated by the manufacturer (30 to 240 bpm), or (2) if temperature decreased by 0.5 °C or more and HR was above its median plus 3 times its median absolute deviation [14,15] during 5 minutes at the end of a measurement period. The latter indicates that the sensor was removed and not directly put on the charger. Evaluation of the quality of measurement data was not part of this study, although earlier work on Everion measurements at the surgical ward showed that 1.2% of HR measurements were excluded for these reasons [12]. Second, a day of HR measurements was excluded from the analysis when less than 1 hour was available on that day. For the daily step count, the minimum available hours were set at 8 hours. Third, patients with less than 7 days of preoperative telemonitoring were excluded from data analyses.

### Statistical Analysis

For each patient, the mean values of HR, step count, and PROMs measured on all included preoperative days were used as individual reference values. Two specific preoperative days were of interest: the first day of telemonitoring and the last day before hospital admission. We hypothesized that the behavior of patients might be different these days, resulting in lower reliability. The intraclass correlation coefficient (ICC) was used to assess the reproducibility between the 2 specific preoperative moments on the one hand and the reference values on the other hand. In addition, mean values of randomly selected measurement periods during the preoperative phase (excluding the first day of telemonitoring and the day before hospital admission) were used to determine the degree to which these measurements provide results similar to the reference values. Randomly selected contiguous periods ranged from 1 to 7 days for both HR and daily step counts, for days with at least 8 hours of HR measurements. For HR, randomly selected periods of 1 hour and 4 hours were used as well. For PROMs, ICCs were computed for one single randomly selected measurement.

The ICC, with its 95% CI based on absolute agreement, two-way random, and average measures, was used to evaluate the reliability. An ICC of ≤0.5 indicated poor, between 0.5 and 0.75 moderate, between 0.75 and 0.9 good, and >0.9 excellent reliability [16]. In addition, Bland-Altman plots with the difference against the average of paired values of HR and daily step count from the 2 specific preoperative moments were used to quantify the agreement between these measurements and the reference values. The mean difference (consistent bias) and the 95% limits of agreement (LoA) were estimated as well.

To describe the perioperative course of HR, daily step count, and PROM values over time in the home situation, the mean value per outcome per day was calculated for each patient. A boxplot was used to visualize these values for all patients from 14 days before hospital admission until 30 days after surgery.

## Results

### Study Participants

A total of 20 patients planned for major open abdominal surgery participated in this study and started telemonitoring at home with a median of 25 (IQR 18-45) days before surgery. The median time between being put on the waiting list and study inclusion was 11 (IQR 5.8-24.4) days. In total, 16 patients had at least 7 days of preoperative measurements and were included in the data analyses. Patient characteristics are shown in Table 1.

**Table 1:** Preoperative patient characteristics (n=16).

Descriptive		Values
Age (years), median (IQR)		69 (62.8-73.0)
**Gender, n (%)**		
	Man	13 (81)
	Woman	3 (19)
**American Society of Anesthesiologists’ classification, n (%)**		
	II	8 (50)
	III	8 (50)
**Comorbidities, n (%)**		
	Cardiovascular disease	8 (50)
	Hypertension	5 (31)
	Chronic pulmonary disease	2 (13)
	Renal insufficiency	2 (13)
	Nonsurgery related malignancy	1 (6)
**Surgical procedure, n (%)**		
	Open abdominal aortic aneurysm repair	7 (44)
	Hepatobiliary surgery	4 (25)
	Gastrointestinal surgery	5 (31)

### Individual Reference Values

For the 16 patients, the median number of reference days was 21 (IQR 14.5-38.5) for HR, 18 (IQR 13.5-35.5) for steps, and 18 (IQR 14-40) for PROMs. The number of hours of sensor data per day and reference values per parameter is summarized in Table 2, which also shows this information for the 2 specific preoperative days: the first day of telemonitoring and the day before hospital admission. Individual reference values and mean values on the 2 specific days are shown in Multimedia Appendix 1.

**Table 2:** Information about measurements on the first day of telemonitoring, reference days, and the day before hospital admission, including measured values.

Parameter	First day of telemonitoring	Reference days	Day before hospital admission
Patients with sensor data, n	15	16	11
Number of hours with sensor data per day, median (IQR)	15 (14-15.8)	12.6 (11.6-14.1)	14 (10.8-14.8)
Mean HR^a^ in bpm^b^, median (IQR)	76.2 (65.5-79.6)	73.3 (66.6-80.4)	73.7 (70.6-83.9)
Standard deviation HR in bpm, median (IQR)	7.9 (7-10.4)	9.7 (8.4-11.7)	10.4 (8.9-10.7)
Daily step count by total number, median (IQR)	1645 (662-3696)	2763 (1576-6320)	2819 (1148-5218)
Mean pain on VAS^c^ 0-10, median (IQR)	0.3 (0-2.4)	0.3 (0.1-4.5)	2.1 (0.1-5)
Mean anxiety on VAS 0-10, median (IQR)	0.4 (0.1-2.6)	0.7 (0-2.3)	1.3 (0.2-4.7)
Mean fatigue on VAS 0-10, median (IQR)	0.82 (0.1-2.3)	0.5 (0.1-3.9)	0.8 (0.1-5)
Mean nausea on VAS 0-10, median (IQR)	0.2 (0-2.1)	0.2 (0-1.3)	0.7 (0.1-2.3)

^a^HR: heart rate.

^b^bpm: beats per minute.

^c^VAS: visual analog scale.

### Reliability

Table 3 shows the ICCs (and 95% CI) between the reference values and the mean values of the 2 specific preoperative days for HR, daily step count, and PROMs. The ICCs of the first day of telemonitoring were 0.91 for HR, 0.71 for steps, and at least 0.86 for PROMs (pain, anxiety, fatigue, and nausea), indicating good to excellent reliability. Good to excellent reliability coefficients (ie, ICC>0.75) were also found between these measurements on the day before hospital admission and the reference values, except for daily step count (ICC 0.71, 95% CI 0.21-0.92).

With regard to the mean values of randomly selected measurement periods during the preoperative phase, ICC values ranged from 0.68 to 0.99 for HR and daily step counts (Table 4). As expected, the ICCs increased over a continuous period of time for both. Good reliability point estimates were achieved after measuring at least 1 day. However, a lower bound of the 95% CIs of at least 0.75 (indicating good reliability) was determined using periods of at least 3 days (Table 4).

Randomly selected PROM measurements compared to the reference values resulted in an ICC of 0.93 (95% CI 0.82-0.97) for pain, 0.94 (95% CI 0.84-0.98) for anxiety, 0.91 (95% CI 0.77-0.97) for fatigue, and 0.89 (95% CI 0.73-0.96) for nausea, indicating good to excellent reliability.

**Table 3:** Intraclass correlation coefficients (ICC and 95% CI) between individual reference values and mean values on the first day of telemonitoring and the day before hospital admission.

Parameter	First day of telemonitoring		Day before hospital admission	
	Patients, n	ICC (95% CI)	Patients, n	ICC (95% CI)
Heart rate	15	0.91 (0.76-0.97)	11	0.76 (0.35-0.93)
Daily step count	13	0.71 (0.30-0.90)	10	0.71 (0.21-0.92)
Pain	14	0.86 (0.64-0.95)	10	0.99 (0.95-1.00)
Anxiety	14	0.90 (0.72-0.97)	10	0.92 (0.74-0.98)
Fatigue	14	0.94 (0.83-0.98)	10	0.97 (0.89-0.99)
Nausea	14	0.87 (0.64-0.95)	10	0.89 (0.64-0.97)

**Table 4:** Intraclass correlation coefficients (ICC and 95% CI) between individual reference values and mean values of randomly selected periods for heart rate (HR) and daily step count.

Period	HR		Daily step count	
	Patients, n	ICC (95% CI)	Patients, n	ICC (95% CI)
1 hour	16	0.68 (0.30-0.87)	N/A^a^	N/A
4 hours	16	0.74 (0.41-0.90)	N/A	N/A
1 day	16	0.86 (0.65-0.95)	16	0.78 (0.49-0.92)
2 days	16	0.87 (0.68-0.95)	16	0.85 (0.63-0.94)
3 days	16	0.90 (0.75-0.96)	16	0.92 (0.80-0.97)
4 days	16	0.92 (0.80-0.97)	15	0.97 (0.92-0.99)
5 days	15	0.99 (0.96-0.99)	15	0.97 (0.91-0.99)
6 days	15	0.97 (0.91-0.99)	14	0.97 (0.90-0.99)
7 days	13	0.99 (0.95-1.00)	13	0.99 (0.96-1.00)

^a^N/A: not applicable.

### Agreement

**Figure 2:**
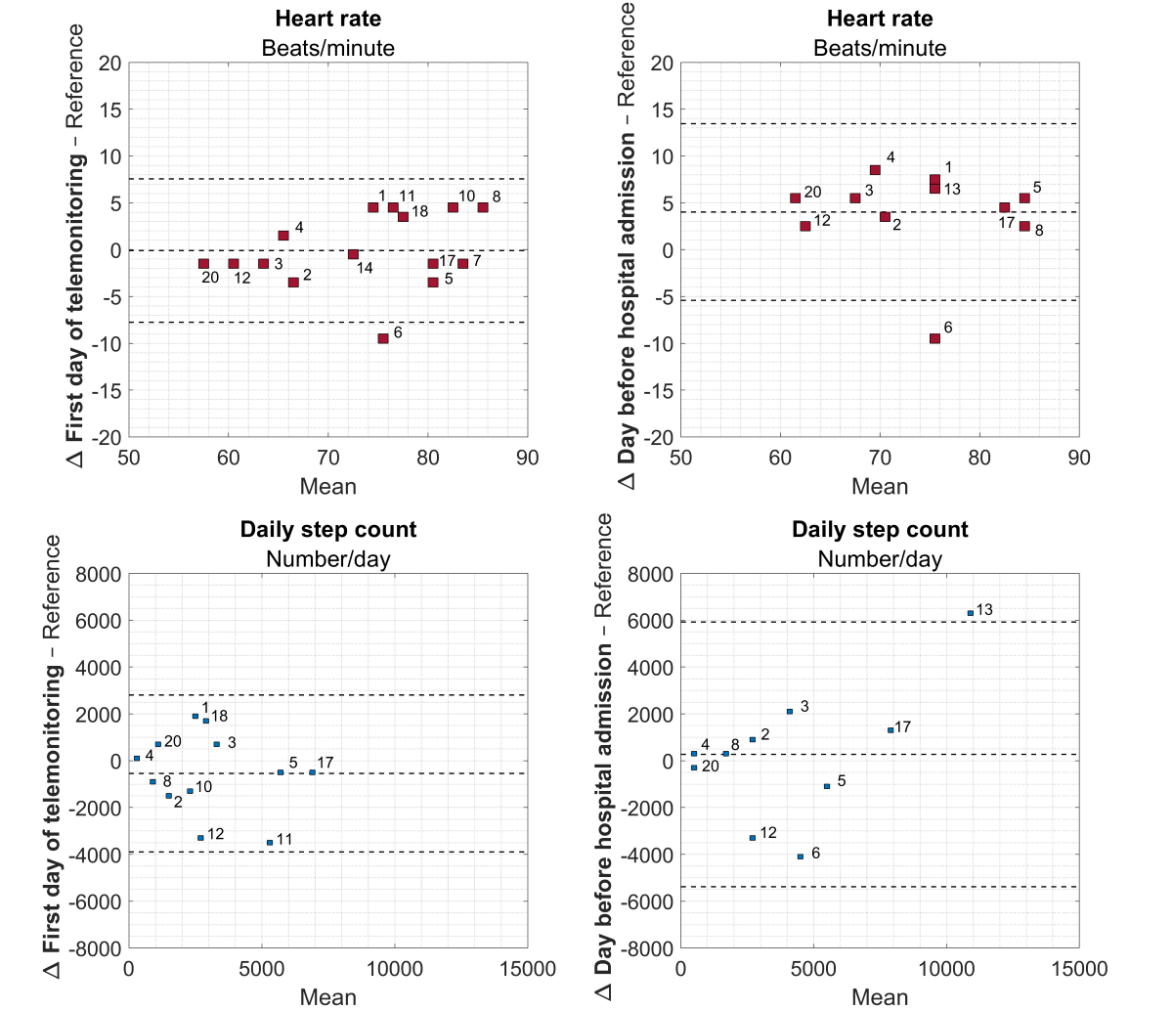
Bland-Altman plots for the mean values per patient for heart rate (upper row) and daily step count (bottom row) on the first day of telemonitoring (left column) and the day before hospital admission (right column). The middle dotted line represents the mean difference and the outer dotted lines the 95% limits of agreement. Numbers represent individual patients.

Bland-Altman plots for HR and daily step count using the reference values and the mean values of the first day of monitoring as well as the day before hospital admission are shown in Figure 2. While there was no bias in the mean HR values on the first day of monitoring (mean difference –0.1, 95% LoA –7.8 to 7.6 bpm), the mean difference (bias) of HR values measured on the day before hospital admission was 4 (95% LoA –5.4 to 13.5) bpm.

The mean difference in daily step counts measured on the first day of telemonitoring was –546 steps with a 95% LoA ranging from –3897 to 2805 steps. This mean difference changed to 270 steps with a broader 95% LoA of –5383 to 5923 steps on the day before hospital admission.

### The Perioperative Course

**Figure 3:**
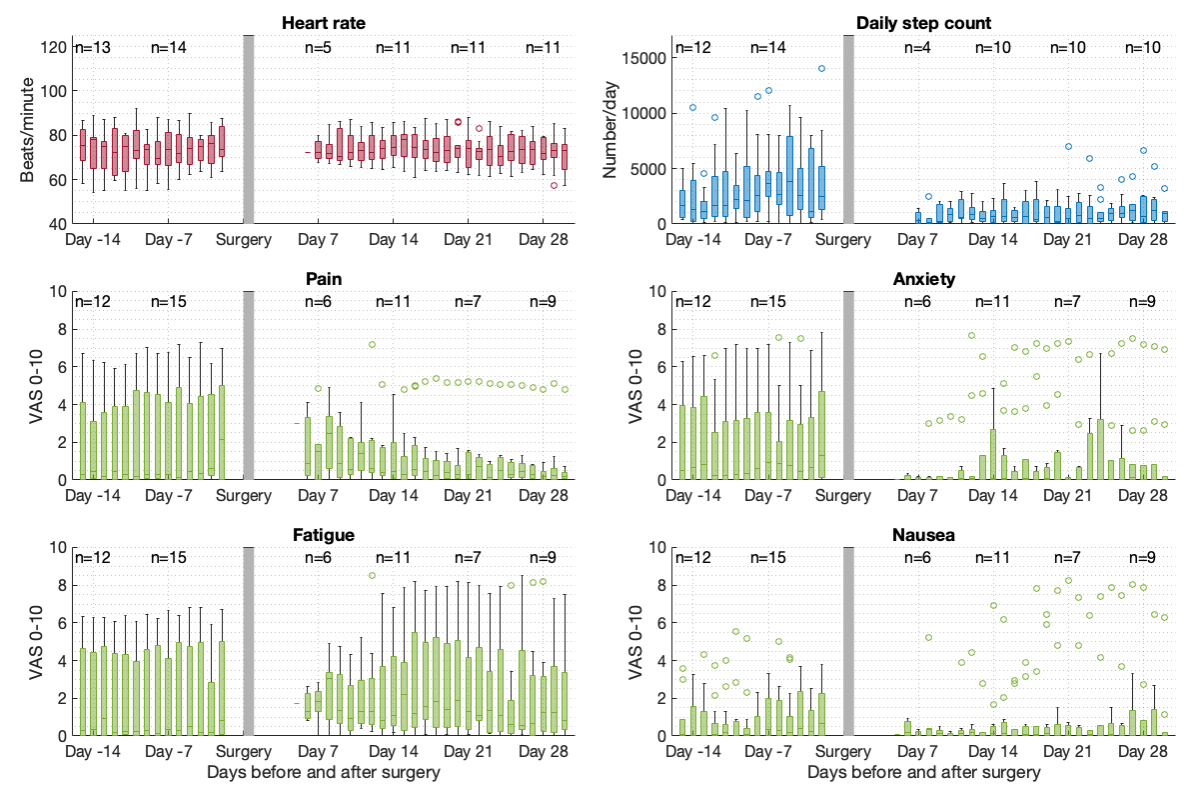
Boxplot of mean values of patients per day for each parameter in the 14 days before hospital admission and 30 days after surgery at home. Boxplots show the median values (bold lines), IQRs (limits of boxes), ranges (whiskers), and outliers (circles). VAS: visual analog scale.

Figure 3 shows the mean values per day measured by telemonitoring at home in the 14 days before and 30 days after surgery for HR, the number of daily steps, and PROMs. Postoperative telemonitoring data at home was available in 13 patients for a median of 17 (IQR 9-21) days. Noticeable is the variability of daily measurements of preoperative steps with a median of 2409 (IQR 1735-4661) steps/day and the lower postoperative step count with a median of 884 (IQR 474-1605) steps/day. In addition, it can be observed that pain reduces over time after surgery at home (not statistically tested).

## Discussion

### Principal Findings

The primary aim of this study was to evaluate during which period patients should be monitored minimally to obtain reliable preoperative baseline values before major open abdominal surgery. Based on the results from this pilot study, a period of 3 days seems to be sufficient for reliable baseline values for HR and daily step count. PROMs had good to excellent reliability on any day, including the first day of telemonitoring and the last day before hospital admission.

The secondary aim was to describe the perioperative course of HR, daily step count, and PROMs measured at home. Visualization of mean values per day mainly showed variable preoperative daily step counts and lower postoperative daily step counts. In addition, pain was visually reduced until 30 days after surgery at home.

### Comparison to Prior Work

Preoperative continuous monitoring of HR in the home environment is currently hardly used or investigated. In a recent study, the resting HR of patients undergoing elective major colorectal surgery was measured during 30 preoperative days with a wearable sensor (Fitbit Charge 2, Fitbit Inc) [8]. The authors found no differences between the mean preoperative HR in patients with or without readmission within 30 days after surgery. Gräfitsch et al [4] asked 16 patients to measure HR twice a day for 7 days before abdominal wall hernia surgery (minor surgery) to generate a baseline. They only reported that the median HR remained stable over the perioperative period. Based on our results, a minimum of 3 days would be sufficient to measure reliable baseline values for HR. However, the clinical implications of these baseline values should be further investigated in future studies.

The daily step count has been mainly objectively measured after surgery and associated with postoperative outcomes [17,18]. Studies that monitored steps before major abdominal surgery show median numbers of 4151 to 4526 [8], 6209 [6], and 6562 [3] daily steps during 30 days, 2 days, and 3 to 7 days preoperatively, respectively. The higher median number of steps compared to our findings (median 2409 steps/day) might be due to the fact that patients in our study were older: median 69 (IQR 62.8-73.0) years versus median 55.5 (IQR 25.5-61.5) years and 58.0 (IQR 42.0-65.0) years [8], mean 55.2 (SD, 11.9) years [6], and median 55.5 (range 22-74) [3] years. Interestingly, we found a mean difference of minus 545 steps between the first day of telemonitoring and the reference values. Although patients were aware of the observational nature of the study, it was expected that the effect of being monitored (due to reactivity [19] and the novelty effect) would have led to a higher step count during the first period of telemonitoring. Optimizing physical activity preoperatively is part of enhanced recovery after surgery and prehabilitation programs, in which wearable sensors are very promising to assist in informing and supporting the patient and clinician [8,20].

PROMs are mostly applied in telemonitoring studies to detect changes during postoperative recovery [21-24]. One study reported a mean VAS of 2.2 (SD 3.1) for pain, 3.1 (SD 2.4) for fatigue, and 0.5 (SD 1.2) for nausea before major abdominal surgery without further use of these values [3]. Preoperative VAS for pain and fatigue reported in our study were lower with median reference values of 0.3 (IQR 0.1-4.5) for pain and 0.5 (IQR 0.1-3.9) for fatigue, while VAS for nausea was comparable with a median of 0.2 (IQR 0-1.3). Even though PROMs are subjective, especially relevant within context (eg, diagnosis and comorbidities), and are moment dependent, our results show that PROMs can be reliably measured on any preoperative day. This creates possibilities for their future use as baseline values, for example, to assess patients’ resilience before surgery and for prehabilitation.

Technological developments enable preoperative evaluation in a patient’s own environment and over a longer period to get more representative individual values. Despite this, practicality and organizational flexibility are also important for application in clinical practice. Although the literature is inconclusive about the minimum period for physical activity measurement, accelerometers are usually worn for up to 7 days, and it is common to include 4 out of 7 days with 10 hours/day wear time, including one weekend day [25]. Moreover, both the first and last measurement days are often omitted [25]. The minimum measurement period of 3 consecutive days of 8 hours to measure HR and daily step count found in our study indicates that effects from daily life activities on sensor measurements are sufficiently averaged during this period.

### Strengths and Limitations

This study provides the first step toward the clinical application of preoperative telemonitoring. One of the strengths of this study is the random selection of measurements to find a reliable period for baseline values. Another strength of this study is the heterogeneity of the patients, which reflects the diversity of the surgical population for which perioperative telemonitoring may be of added value. First, an important limitation of this study is the small number of included patients. Four patients were excluded from data analyses due to the short period of preoperative measurements. The main reasons for this were that patients were scheduled for surgery earlier due to program dropout or connectivity problems. However, the choice of the minimum length of the reference period as well as the exclusion criteria for the minimum number of available data points per day was arbitrary as this is one of the first times preoperative baseline values derived by telemonitoring have been investigated. In the future, this could be improved by using a larger study population and refining the criteria to exclude data periods. Second, another limitation is that of all the vital signs measured by the wearable sensor, only HR was included in this study because the validity and reliability of the sensor for respiratory rate, blood oxygen saturation, and temperature were low [11,12]. Third, the validity and reliability of the sensor for daily step count are still unknown, which limits the translatability of the current results to other wearable sensors that measure daily step count. 

### Future Directions

Resting HR and HR during physical activity were not investigated in this study because activity parameters were stored once per hour and the sensor was not worn during the night due to charging. This could provide additional information and may be taken into account in future research.

Preoperative measurements of vital signs, physical activity, and PROMs may be used in future studies regarding prehabilitation or personalized monitoring of the entire perioperative period. In general, knowledge about the association between these parameters at home is scarce. For example, it is known that HR is highly affected by physical activity, and pain has been associated with decreases in daily steps [26]. A larger observational study monitoring vital signs, physical activity, and PROMs in surgical patients might be useful in understanding these associations since they are relevant for the interpretation of the telemonitoring data in clinical practice.

The generalizability of these results is limited due to the small sample size and limitations of the used sensor. However, this was a pilot study to assess the feasibility of perioperative telemonitoring [9] and to get an idea of the required period to measure preoperative values to inform future study design. The used method in this study could be applied to find a measurement period for reliable estimation of baseline values of other continuously monitored vital signs, patient populations, and wearable sensors as well.

### Conclusions

In patients awaiting major abdominal surgery, baseline values for HR and daily step count could be measured reliably by a wearable sensor worn for at least 3 consecutive days in this study. PROMs could be measured with good to excellent reliability on any given day, including the first day of telemonitoring and the day before hospital admission. Visualization of mean values of HR, step count, and PROMs on the days before and after major abdominal surgery at home provided insight into the perioperative course of [these parameters in] our study population, although no clear conclusions could be drawn from this. Future work should focus on the clinical implications of these baseline values.

## Appendix

Multimedia Appendix 1 Mean values on the first day of telemonitoring, the reference values, and mean values on the day before hospital admission for each parameter per patient. Each color corresponds with an individual patient. VAS: visual analog scale.

## Abbreviations

bpm: beats per minute
HR: heart rate
ICC: intraclass correlation coefficient
LoA: limits of agreement
PROM: patient-reported outcome measure
PROMISE-study: Telemonitoring in the Peri-operative Phase of Patients Undergoing Open Abdominal Surgery in a University Medical Center: A Pilot Study
STROBE: Strengthening the Reporting of Observational Studies in Epidemiology
VAS: visual analog scale

## Data Availability

The data sets generated and analyzed during this study are available from the corresponding author on reasonable request.
