# Supplementary figures and images for: Determining the Reliable Measurement Period for Preoperative Baseline Values With Telemonitoring Before Major Abdominal Surgery: Pilot Cohort Study
Source: JMIR Perioper Med. 2022 Nov 28;5(1):e40815. doi: 10.2196/40815 (PMC9745646; doi:10.2196/40815)

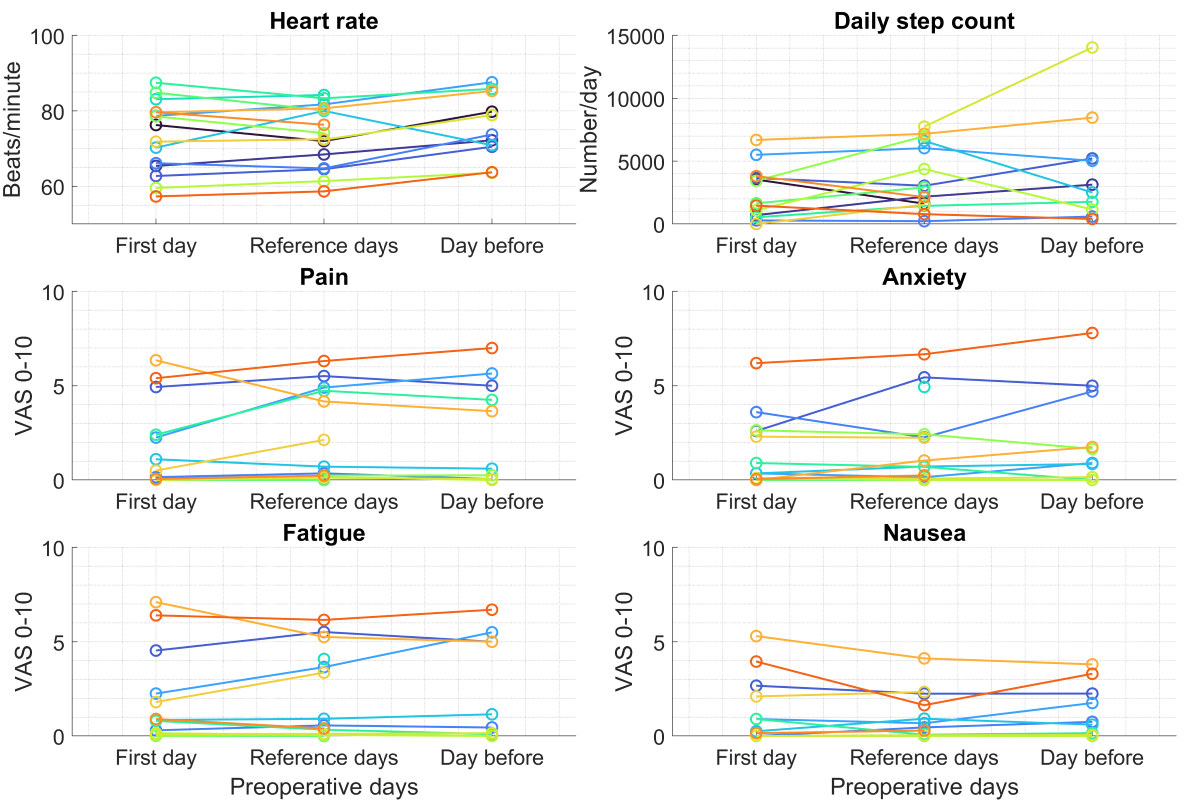

Supplement: Multimedia Appendix 1 [file periop_v5i1e40815_app1.png]
